# Improving the Antioxidant Potential of Berry Crops Through Genomic Advances and Modern Agronomic and Breeding Tools

**DOI:** 10.3390/biotech14040089

**Published:** 2025-11-07

**Authors:** Georgios Mitronikas, Athina Voudanta, Aliki Kapazoglou, Maria Gerakari, Eleni M. Abraham, Eleni Tani, Vasileios Papasotiropoulos

**Affiliations:** 1 Laboratory of Plant Breeding & Biometry, Department of Crop Science, Agricultural University of Athens, Iera Odos 75, 11855 Athens, Greece; gio.mhtronikas@hotmail.com (G.M.); athinavoudanta@gmail.com (A.V.); mgerakari@aua.gr (M.G.); etani@aua.gr (E.T.); 2 Institute of Olive Tree, Subtropical Crops and Viticulture (IOSV), Department of Vitis, Hellenic Agricultural Organization-Dimitra (ELGO-Dimitra), Sofokli Venizelou 1, Lykovrysi, 14123 Athens, Greece; kapazoglou@elgo.gr; 3 Laboratory of Range Science, School of Agriculture, Forestry and Natural Environment, Aristotle University of Thessaloniki, 54124 Thessaloniki, Greece; eabraham@for.auth.gr

**Keywords:** antioxidants, berry crops, breeding, genomic tools, grapevine, nutritional value

## Abstract

The growing demand for sustainable, health-promoting foods has intensified efforts to improve the antioxidant potential of berry crops through integrative agronomic, genomic, and breeding innovations. Berries are rich dietary sources of bioactive compounds that support human health and provide benefits far beyond basic nutrition. This review explores the diversity of major berry crops, including blueberries, raspberries, cranberries, blackberries, and grapes, with emphasis on their nutritional value and antioxidant profiles. It also examines their domestication history, wild relatives, and commercial cultivars, offering insight into the genetic and phenotypic diversity underlying their rich chemical composition. Furthermore, the review highlights the application of modern tools to enhance antioxidant content. By integrating agronomic practices such as seed priming and grafting, advanced molecular breeding technologies, including multi-omics, genome-wide association studies (GWAS), and genome editing, breeders and researchers can accelerate the development of high-value berry cultivars that combine superior nutritional quality, resilience to environmental stress, and sustainable productivity under the challenges posed by climate change.

## 1. Introduction

### 1.1. A Multitude of Berry Crops

Berry crops, including grapes, comprise a diverse group of fruits that differ botanically yet share similar cultivation practices, postharvest behavior, and health-promoting properties [1]. A true berry is a simple fruit that develops from a single ovary and contains multiple seeds within a fleshy pericarp, such as blueberries (*Vaccinium* spp.), cranberries (*Vaccinium macrocarpon*), and grapes (*Vitis vinifera*) [2]. In contrast, fruits such as raspberries (*Rubus idaeus*) and blackberries (*Rubus fruticosus*), though commonly referred to as berries, are in fact aggregate fruits, consisting of numerous small drupelets that originate from a single flower with multiple ovaries [3]. Despite this structural difference, all these fruits are commonly grouped under “berry crops” due to their overlapping agricultural, nutritional, and commercial significance [4].

Among major cultivated berries, raspberries, native to Europe and northern Asia, yield about 940,000 t globally, with Russia, Mexico, Serbia, Poland, and the United States as leading producers. Blueberries, native to North America, total about 1.1 million t, dominated by the United States, Peru, Canada, Chile, and Spain. Cranberries, also of North American origin, produce around 470,000 t, largely from the United States, Canada, and Chile. In contrast, grapes, native to the Near East, far exceed berry crops with over 72.5 million t, produced mainly in China, Italy, Spain, the United States, and France [5] (Table 1).

Berry cultivation reflects a combination of historical domestication patterns, ecological adaptations, and modern agricultural practices. Raspberries, blackberries, blueberries, and cranberries are primarily associated with temperate or specialized agro-ecological zones, whereas grapes represent one of the most extensively cultivated fruit crops worldwide. A comparative overview of their production statistics, major producing countries, and key soil–climate requirements is presented in Table 1.

### 1.2. Nutritional and Antioxidant Value of Berries as Pharmaceutical and Nutraceutical Sources

Various human diseases are linked to free radicals, with risks increased by lifestyle factors, pollution, smoking, drugs, and stress [6,7]. Antioxidants, found naturally in fruits like berries and other foods, scavenge free radicals, reducing oxidative damage and protecting against premature aging, cardiovascular disease, and degenerative disorders such as cataract, Alzheimer’s disease, and cancer [8]. They also exhibit antihypertensive, antimicrobial, anti-inflammatory, anti-fibrotic, and anticancer activities [9]. Moreover, beyond these effects, antioxidants play a central role in maintaining overall human health by preserving cellular integrity, supporting immune function, and modulating inflammatory pathways [10]. Diets rich in natural antioxidant-bearing phytochemicals from plant sources contribute to redox homeostasis and are consistently associated with enhanced metabolic health and a lower risk of chronic disease development [11]. For centuries, fruits such as berries have been valued in traditional medicine and human diets for their health-promoting properties. Despite limited scientific understanding, their historical use was based on empirical knowledge; however, advances in phytochemistry, particularly through metabolomics, have revealed the complex mixture of bioactive compounds responsible for their beneficial effects on human health [12].

For centuries, fruits such as berries have been valued in traditional medicine and human diets for their health-promoting properties. Despite limited scientific understanding, their historical use was based on empirical knowledge; however, advances in phytochemistry, particularly through metabolomics, have revealed the complex mixture of bioactive compounds responsible for their beneficial effects on human health [13].

Berries are an abundant source of antioxidants, particularly flavonoids. Among these, flavonols, such as quercetin and kaempferol, present in raspberries, cranberries, and grapes have been reported to significantly contribute to their antioxidant capacity and associated health benefits [13]. Quercetin, present in raspberries and cranberries, demonstrates significant anti-inflammatory, antiviral, and antihistamine activities and has been studied as an supportive therapy for arthritis, allergies, and viral infections [14,15,16].

Anthocyanins, like cyanidin-3-glucoside (C3G), malvidin-3-glucoside (M3G), peonidin-3-glucoside (P3G), and delphinidin-3-glucoside (D3G) are highly concentrated in blueberries, blackberries, and red or purple grapes, where they are primarily responsible for the characteristic pigmentation [14]. Beyond coloration, anthocyanins exhibit strong neuroprotective and cardioprotective effects, contributing to the prevention of neurodegenerative diseases such as Alzheimer’s and Parkinson’s, enhancement endothelial function, and reduction in blood pressure [17,18]. Grapes and cranberries are rich in flavan-3-ols, particularly catechin and epicatechin, which are concentrated in the seeds [19]. According to the USDA Database for the Flavonoid Content of Selected Foods (Release 3.3), antioxidant composition varies both qualitatively and quantitatively among berry species. Blackberries and blueberries are rich in cyanidin, delphinidin, and malvidin, while raspberries predominantly contain cyanidin at lower concentrations. Cranberries are characterized by high levels of flavan-3-ols (catechin and epicatechin) alongside anthocyanins, whereas grape flavonoid profiles depend on color red and black cultivars accumulate anthocyanidins, while white grapes primarily contain flavan-3-ols with only trace anthocyanins [20].

Stilbenoids, such as resveratrol and pterostilbene, are a group of phenolic compounds, mainly present in red and purple grapes, exhibit multifunctional antioxidant and anti-inflammatory properties through modulation of oxidative and inflammatory pathways and are linked to cardioprotective effects [21].

Tannins are also well represented in berry crops. Hydrolysable tannins such as ellagic acid are abundant and associated with cardiovascular protection, antimicrobial and antiviral activity, anti-inflammatory effects, and potential anticancer properties [22]. Condensed tannins (proanthocyanidins) are predominant in grape seeds, cranberries, and blackberries, contributing further to their bioactivity. Proanthocyanidins, particularly A-type forms, which are abundant in cranberries, are known for inhibiting bacterial adhesion in urinary tract infections and increasingly investigated for their roles in gut microbiome modulation and vascular health [23].

Tannin profiles differ both qualitatively and quantitatively among species. Raspberries predominantly synthesize hydrolysable tannins (ellagitannins), whereas blueberries mainly contain condensed tannins (proanthocyanidins), with minimal hydrolysable forms. Raspberries exhibit higher total ellagitannin levels (up to 326 mg/100 g FW), while blueberries show moderate tannin contents (≈160 mg/100 g FW), largely composed of highly polymerized proanthocyanidins (degree of polymerization > 10) [24]. These variations reflect species-specific metabolic specialization influenced by tissue type, developmental stage, and environmental conditions.

Phenolic acids, including chlorogenic, caffeic, and gallic acids, are widely distributed in blueberries, grapes, and cranberries [20], where they exhibit antiviral activity, regulate glucose metabolism, improve insulin sensitivity, and contribute to diabetes and obesity prevention [21]. Species differ markedly in phenolic composition: raspberries are rich in ellagitannins and ellagic acid derivatives; blueberries in hydroxybenzoic and hydroxycinnamic acids; blackberries in chlorogenic- and ferulic-type acids; grapes in cultivar-dependent mixtures of phenolic acids and anthocyanins; and cranberries in A-type proanthocyanidins. Quantitative differences are also notable; blackberries average 248 mg/100 g, blueberries 525 mg/100 g, cranberries 120–315 mg/100 g, raspberries 126 mg/100 g, and grape seeds up to 500 mg/100 g of total phenolics [25], reflecting genetic, metabolic, and structural specialization across species [26,27,28,29]. This biochemical diversity underpins the distinct health-promoting potential and functional properties of each berry species.

Overall, the diversity of flavonoids, phenolic acids, tannins, and other bioactive compounds among berry species arises from genetic, environmental, and developmental regulation of the phenylpropanoid and flavonoid biosynthetic pathways. Species- and cultivar-specific metabolic control determines dominant phenolic subclasses, such as ellagitannins in raspberries, hydroxycinnamic acids in blueberries, and proanthocyanidins in blackberries [26,27,29]. Environmental conditions, fruit pigmentation, tissue type and developmental stage further modulate compound distribution [24,28]. These interacting factors account for the substantial qualitative and quantitative variation observed in the bioactive profiles across berry and grape species.

**Figure 1:**
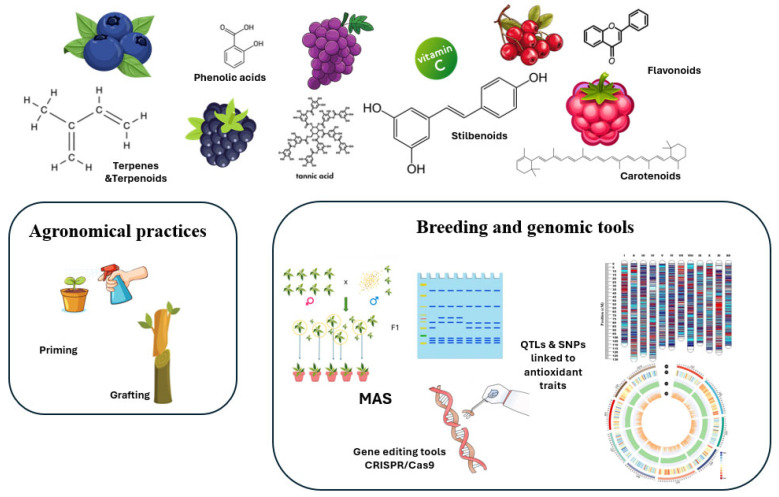
Exploitation of berry crops antioxidant capacity through agronomical practices and molecular breeding methods utilization.

Minor bioactive compounds, such as vitamins C and E, carotenoids (β-carotene, lutein), and terpenes (limonene, myrcene, linalool, pinene) enhance cellular antioxidant defenses, neutralize free radicals, and mitigate inflammatory stress [30]. Additionally, essential minerals including calcium, magnesium, and iron contribute to antioxidant defense and metabolic homeostasis [31,32]. Calcium and magnesium support enzymatic activity and oxidative stress regulation, while iron, though required in small amounts, is crucial for oxygen transport and redox balance and interacts synergistically with polyphenols and vitamin C [33]. This combined action of antioxidants, vitamins, and micronutrients underscores the nutritional and functional value of berries in reducing inflammation and protecting against chronic diseases [34]. Based on the recognized nutritional and pharmaceutical value of the antioxidants of berries, current research is directed toward tools that can increase their accumulation in fruits. In this context, agronomical techniques, such as priming and grafting, can stimulate antioxidant pathways by improving plant resilience under stress conditions. At the same time, molecular breeding approaches, including marker-assisted selection, QTL mapping, and genome editing, offer targeted opportunities to enhance antioxidant biosynthesis. The integration of these strategies provides a comprehensive framework that connects crop improvement with the development of berry fruits enriched in antioxidants and enhanced health benefits (Figure 1).

## 2. Agronomical Practices for Increasing Antioxidant Concentration

### 2.1. Priming

Environmental changes have intensified abiotic stress in plants, reducing yield and threatening food security [35]. One adaptive mechanism that improves plant resilience is priming, a process that prepares plants to respond more efficiently to future stresses [36].

Priming, also referred to as sensitization or hardening, induces a “primed state” (PS) that enhances the plant’s ability to activate defense mechanisms when challenged again [37]. This state is triggered by mild pre-exposure to stress or by the application of natural or synthetic agents. Biochemical mechanisms underlying PS include the accumulation of inactive signaling proteins or transcription factors, activation of mitogen-activated protein kinases (MAPKs), changes in redox status, epigenetic modifications, and breakdown of certain cellular compounds [38]. Once primed, plants exhibit enhanced redox balance and faster stress responses, improving resilience and growth [39].

Notably, priming strategies have been shown to enhance the antioxidant capacity of berry crops, thereby improving both stress resilience and nutritional quality. For instance, in blueberries, foliar application of polyamines such as putrescine and spermidine significantly increased antioxidant potential, with the fruit development stage identified as the most favorable period for treatment. Such findings provide valuable guidance for growers to optimize fertilization practices during critical phases of berry growth and maturation [40]. Likewise, in blackberries, priming with *Bacillus amyloliquefaciens* has been used to investigate the role of the flavonol–anthocyanin pathway in the adaptation of plants to biotic stress. This treatment modulated blackberry metabolism and identified key target genes, providing insights for improving plant adaptability and fruit quality [41]. Additionally, a biostimulant derived from *Ascophyllum nodosum*, applied as a foliar spray to raspberry, was found to enhance yield by over 30% while maintaining most nutritional traits. Notably, magnesium content increased in primed fruits, highlighting its potential as a sustainable tool to improve berry yield and quality [42]. Similarly, pre-harvest foliar treatments with ethephon (Eth) at the ‘veraison’ stage resulted in increased levels of total phenolics and anthocyanin and enhanced antioxidant capacity accompanied by an increased expression of the anthocyanin biosynthesis genes, such as *chalcone synthase* (*CHS*), *flavanone 3-hydroxylase* (*F3H*), and *flavanol 3-o-glucosyl transferase* (*UFGT*) in ‘Crimson Seedless’ grape berry skins [43]. Moreover, postharvest treatment with melatonin reduced grape berry abscission and decay, enhanced amino acid accumulation, and activated endogenous melatonin and phenolic biosynthesis pathways, highlighting the potential for improving postharvest quality through the modulation of secondary metabolism [44].

### 2.2. Grafting

Grafting, a technique that is widely used in perennial crops, has been adopted more in berry species such as raspberries and blueberries with the aim of improving the plant’s stress tolerance and fruit quality [45]. By combining the root system of a robust rootstock with the scion of a high-yielding or high-quality cultivar, growers can modify physiological traits in a manner that is not possible through conventional breeding. Even though grafting is more commonly linked to disease resistance and soil adaptability, recent studies indicate it may also affect the antioxidant capacity of berry fruits. Changes in nutrient uptake, water-use efficiency, and hormonal signaling can result from physiological interactions between the rootstock and scion. These changes are closely related to the fruit’s generation of secondary metabolites [46].

The accumulation of phenolic compounds, anthocyanins, and flavonoids can be indirectly enhanced by specific rootstocks, according to evidence from grafted raspberry and blueberry systems [45]. Increased photosynthetic activity, a more stable water balance during drought, and improved root-to-shoot nutrient transport, particularly minerals like magnesium and zinc that are essential in antioxidant enzyme function, are frequently linked to these gains [47].

Specifically for grapes, grafting has notably improved antioxidant capacity by adjusting the scion’s physiological and biochemical reactions through rootstock selection. According to a study by Krishankumar et al. [48], the use of drought-tolerant rootstocks, like Ramsey, R110 and 1103 Paulsen, positively resulted to a significant increase in important antioxidant enzyme activities, including glutathione reductase (GR), ascorbate peroxidase (APX), peroxidase (POD), catalase (CAT), and superoxide dismutase (SOD), under drought stress. Furthermore, Klimek et al. [49], showed that different rootstocks significantly affected the content of bioactive compounds in Regent grapes. The DPPH radical scavenging measurement in grafted grapes revealed variations in L-ascorbic acid, phenolic acids, total flavonoids, anthocyanins, and tannins, all of which are directly related to antioxidant activity. Together, these findings prove that choosing the right rootstocks greatly increases the antioxidant profile and nutritional value of grapes in addition to increasing their resistance to abiotic stress.

## 3. Berry Genomics and Breeding for Enhancing Antioxidant Capacity

Berries have gained recognition not only for their delightful taste but also for their potential health benefits. However, to ensure that these benefits reach consumers, it is essential to enhance fruit quality throughout the entire production chain [50]. The nutritional quality of berry crops is a multidimensional trait, determined by agronomic productivity, organoleptic attributes, and chemical composition, including antioxidant content. Historically, breeding programs have prioritized yield, fruit appearance, and postharvest shelf life as the main drivers of market success. While these selective pressures have improved visual appeal and storability, they have sometimes led to unintended, detrimental effects on aroma, flavor, and nutraceutical properties, such as antioxidant content [51].

According to the literature, breeding programs targeting improved tolerance of berries to abiotic stresses such as extreme temperatures, drought, salinity, and iron deficiency have also contributed significantly to enhancing their antioxidant capacity [52]. Building on these health-promoting characteristics, improving the antioxidant content is becoming a central breeding target, guiding current efforts to combine nutritional quality with agronomic performance [53,54]. To achieve these goals, modern breeding strategies integrate advanced genomics and molecular tools [55]. Until now, conventional breeding has been carried out mainly through time-consuming, controlled crossings between selected parents, followed by long-term field evaluations of berry seedlings for traits such as yield, vigor, disease resistance, and fruit quality. Then promising genotypes are clonally propagated to establish new cultivars [55,56].

At the genomic level, high-quality reference genomes have been assembled for several berry crops, facilitating the discovery of gene families involved in secondary metabolite biosynthesis [57,58]. Multi-omic studies have elucidated metabolic pathways and the expression dynamics of regulating genes in response to both developmental and environmental stimuli. Integrated transcriptomic and metabolic profiling reveals that phenolic acid and flavonol biosynthesis in *Rubus chingii* Hu (Chinese raspberry) begins at the onset of fruit development. Afterwards, these compounds are systematically accumulated or metabolized into related derivatives [59]. The exploitation of pathways such as those for the phenylpropanoid or the flavonoid biosynthesis is essential to antioxidant production and is tightly coupled with responses to abiotic stresses in berry crops like blackberry [41].

Moreover, Genome Wide Association Studies (GWAS) have improved trait mapping in berries through investigations of the heritability of quality traits and their associations with known QTLs [60]. At the same time, berry breeding increasingly integrates conventional methods with modern molecular approaches, and CRISPR/Cas9-mediated gene editing has already been validated in blueberries, paving the way for precise trait improvement, including antioxidant enhancement without yield penalties [61].

Metabolic engineering offers powerful approaches to enhance antioxidant biosynthesis in berries and grapevine by targeting the flavonoid and anthocyanin pathways. In *Vaccinium* species, key structural genes such as *anthocyanidin synthase* (*ANS*) and *UDP-glucose: flavonoid 3-O-glucosyltransferase 2* (*UFGT2*) have been shown to regulate anthocyanin accumulation and composition. These genes are essential for the final steps in anthocyanin biosynthesis and manipulating them could lead to new blueberry varieties with enhanced nutritional value [62]. The establishment of a CRISPR/Cas9 editing system to knock out the *phytoene desaturase* gene in highbush blueberry provided proof of concept for gene editing in woody, polyploid plants and created a platform for the precise modification of antioxidant-related genes [63].

Synthetic biology applies engineering principles to crops through iterative “design–build–test–learn” cycles, enabling precise reprogramming of genetic circuits to enhance productivity. By redesigning biological components and systems, it aims to improve yield, resilience, and nutritional quality in plants [64]. Although full synthetic biology approaches, such as modular promoter design, orthogonal regulatory networks, or de novo pathway reconstruction have not yet been reported in berries or grapevine, their feasibility in plants has been demonstrated. Recent advances highlight plants as promising chassis for modular biosynthesis of industrially important natural products, such as terpenoids, alkaloids, and phenylpropanoids, which have applications in medicine, food, and other sectors and emphasize the role of synthetic biology–AI integration for crop metabolic design [64,65]. These developments offer a conceptual framework for future design-based enhancement of antioxidant metabolism in berry and grapevine species.

### 3.1. Blueberry (Vaccinium corymbosum *L*.)

#### 3.1.1. Domestication and Wild Relatives

Blueberry is a widely cultivated native fruit in North America [66]. Domestication began in the early 20th century through extensive interspecific hybridization programs [67]. The genus *Vaccinium* evolved in eastern North America, with biogeographic origins centered in the Appalachian Mountains and Atlantic coastal plains, where natural populations exhibit maximum genetic diversity [68]. Evolutionary relationships within section *Cyanococcus* reveal three main lineages: (1) northern species (*V. corymbosum*, *V. angustifolium*), (2) southern species (*V. darrowii*, *V. tenellum*, *V. virgatum*), and (3) western species (*V. ovalifolium*, *V. deliciosum*) [69]. Polyploidy evolution is prominent, with diploid (2*n* = 2*x* = 24), tetraploid (2*n* = 4*x* = 48), and hexaploid (2*n* = 6*x* = 72) cytotypes, where allopolyploidy has driven speciation and adaptive radiation across diverse ecological niches [70,71]. Cultivated highbush blueberry (*V. corymbosum*) is tetraploid (2*n* = 4*x* = 48), with a monoploid genome size of ~2.53 pg [70,72]. Other cultivated groups include lowbush (*V. angustifolium*, diploid) and rabbiteye (*V. virgatum*, hexaploid), with extensive interspecific hybridization among them [73].

Wild blueberry species harbor broad genetic diversity with desirable traits that can be exploited in breeding programs. While most studies on their genetic variability do not directly assess antioxidant capacity, they often provide indirect insights, such as the presence and stress-induced accumulation of metabolites and flavonoids, that are increasingly targeted to enhance the antioxidant potential of cultivated blueberries [74]. More specifically, wild *Vaccinium* species have been recognized as tolerant genetic material with high nutritional value against various abiotic and biotic stressors, such as low chilling requirements in *V. darrowii* [75], and heat and drought tolerance in *V. myrsinites* and *V. darrowii* [76]. Traits like fruit firmness, late ripening, and disease resistance are well-documented in *V. virgatum* [67]. Moreover, diploid southern species such as *V. tenellum*, *V. elliottii*, and *V. myrsinites* contribute specialized metabolites and stress tolerance [76]. Furthermore, *V. angustifolium,* known as wild lowbush blueberry and native to northeastern North America, has been recognized as a source of cold hardiness and unique flavonoids [77]. High-quality genome assemblies are now available for *V. darrowii* (~1.06 Gb, >34,000 protein-coding genes) and *V. corymbosum* (‘W8520’ assembly, ~393 Mb), providing essential resources for elucidating the genetic basis of antioxidant capacity in berries and for uncovering the molecular mechanisms underlying its biosynthesis [71,75].

Blueberry commercial cultivation focuses on three main groups with distinct antioxidant profiles: (a) Northern Highbush (*V. corymbosum*) cultivars dominate global production and include early-season genotypes such as ‘Duke’ and ‘Rubel’ known for high anthocyanin content and excellent antioxidant capacity [71], and mid-season standards like ‘Bluecrop’ and ‘Frienship’, recognized for a balanced anthocyanin profile and high phenolic diversity [78]. Late-season cultivars such as ‘Elliott’, are distinguished by elevated proanthocyanidin content and extended shelf-life due to antioxidant stability, high total phenolic content and superior ORAC (Oxygen Radical Absorbance Capacity) values [79,80]. According to Stevenson & Scalzo [81], several Northern Highbush cultivars such as ‘Rubel’, ‘Duke’, and ‘Elliott’ consistently exhibit elevated anthocyanin and total phenolic content, reinforcing their value as benchmark genotypes for antioxidant potential. (b) Southern Highbush (*V. corymbosum* × *V. darrowii*) hybrids include ‘Jewel’, a mid-season genotype with elevated flavonol content and enhanced UV protection compounds [67], ‘Legacy’ a mid- to late genotype with high total anthocyanins and phenolics and ‘Emerald’, which features a low-chill requirement and unique anthocyanin glycosylation patterns introgressed from *V. darrowii* [79]. (c) Rabbiteye includes early and late-season harvests cultivars, like ‘Tifblue’ which contribute further diversity, serving as industry standards due to their high tannin content and antioxidant stability [67,80,82]. Late-ripening cultivars such as ‘Velluto Blue’ and ‘Centra Blue’ exhibit the highest fruit yields, anthocyanin contents, and estimated total anthocyanin harvestable from a given area [79]. Additionally, Rabbiteye cultivars, such as ‘Ono’, ‘Centurion’, and ‘Southland’, have been highlighted for their superior antioxidant richness, often surpassing both highbush groups in anthocyanin and phenolic content [81]. All the above-mentioned cultivars represent promising candidates for breeding programs focused on phytochemical enhancement regarding antioxidant properties.

#### 3.1.2. Modern Genomic and Breeding Tools for Enhancing the Antioxidant Profile of Blueberries

Several genes and gene families have been studied in relation to the antioxidant capacity of blueberries, and their roles have been linked to genotypes with desirable traits and high nutritional value. For instance, the MATE gene family, which encodes transport proteins that facilitate the movement of anthocyanins across cell membranes during fruit ripening, has been characterized as *V. corymbosum* [83]. Additionally, genes encoding proteins involved in the flavonoid biosynthetic pathway, such as chalcone synthase (CHS), chalcone isomerase (CHI)*,* flavanone 3-hydroxylase (F3H), flavonoid 3′-hydroxylase (F3’H), flavonoid 3′,5′-hydroxylase (F3’5’H), dihydroflavonol 4-reductase (DFR), anthocyanidin synthase (ANS), UDP-glucose:flavonoid 3-O-glucosyltransferase (UFGT), and leucoanthocyanidin reductase (LAR), are reported to be upregulated during fruit ripening and to correlate with anthocyanin accumulation [74,75]. Notably, *VcGSTF* genes show high expression during ripening and are implicated in anthocyanin stabilization and vacuolar transport [84]. A genome wide analysis of the Caffeic Acid O-Methyltransferase (COMT) gene family was conducted in *V. corymbosum*, identifying 92 *COMT* genes involved in phenylpropanoid metabolism. Moreover, other gene families encoding enzymes involved in the phenylpropanoid pathway, such as phenylalanine ammonia lyase (PAL), anthocyanidin reductase (ANR), cinnamate-4-hydroxylase (C4H), 4-coumarate:CoA ligase (4CL), flavonol synthase (FLS), hydroxycinnamoyl-CoA:shikimate hydroxycinnamoyl transferase (HCT), have also been identified under stress conditions and are associated with antioxidant responses [85,86]. Terpene synthases (TPSs) have also been characterized in blueberries, contributing to volatile profiles and secondary metabolite diversity relevant to antioxidant potential [87]. In the study of Guo et al. [88], fourteen *F3′5′H* genes were identified in the blueberry genome. The authors demonstrated that these genes, particularly *VcF3′5′H4*, are highly expressed during fruit ripening and play a key role in regulating anthocyanin biosynthesis, light response, and secondary metabolism, confirming their importance in pigment formation and stress adaptation.

Table 2 summarizes the genes and their corresponding enzyme classes investigated in blueberries with respect to antioxidant capacity. Despite the availability of high-quality genome assemblies, the precise roles, annotations, and functional characterizations of numerous genes in blueberry remain unresolved and continue to be active areas of research.

Advanced omic approaches such as transcriptome sequencing (RNA-seq) offer powerful insights into antioxidant-related genes, their expression dynamics, and their contribution to key metabolic traits in berry crops. RNA-seq analysis of 196 F1 genotypes, developed from the crosses of the cultivars ‘Draper-selection-44392’ and ‘Jewel’, differing in anthocyanin glycosylation and acylation identified several differentially expressed genes (DEGs). For the major-effect QTL on chromosome 4, three strong candidate genes were detected controlling glycosylation, including the UDP-glucosyltransferase (*Vcev1_p0.Chr04.11988)* and two endo transglycosylation (*Vcev1_p0.Chr04.11996* and *Vcev1_p0.Chr04.11998*). Among them, *Vcev1_p0.Chr04.11988* was significantly up-regulated in high-glycosylated genotypes and validated by qRT-PCR, supporting its role as a key regulator of anthocyanin glycosylation. For acylation, a major locus on chromosome 2 contained two acyltransferase genes (*Vcev1_p0.Chr02.03371* and *Vcev1_p0.Chr02.03383*), both up-regulated in high-acylated genotypes glycosylation and acylation identified several differentially expressed genes (DEGs) [92].

The chromosome-scale, haplotype-resolved assembly of tetraploid highbush blueberry (Draper’) provided a framework for annotating candidate genes, including *UGTs*, genes for acyltransferases, and *HCT*, many colocalize with antioxidant QTLs [71]. Multi-year QTL mapping in a *V. corymbosum* var. *caesariense* × *V. darrowii* population identified a major chlorogenic acid locus on Vc02 overlapping *HCT/UGCT*, and 67 QTLs for anthocyanins across Vc01–Vc12 explaining 11–67% of variance. Major hotspots included 34 QTLs on Vc04 (~115 cM) linked to glucoside-enriched anthocyanins and 15 QTLs on Vc01 (18–35 cM) linked to reduced arabinose/galactose conjugates, with additional loci on Vc02 and Vc08–Vc12 [93]. GWAS with an 89K SNP array identified 47 SNP–trait associations for flavonoid metabolites, revealing novel biosynthetic genes and regulatory networks shaping antioxidant diversity [92]. These studies highlight the polygenic control of antioxidant metabolism in the polyploid blueberry.

### 3.2. Red Raspberry (Rubus idaeus *L*.)

#### 3.2.1. Domestication and Wild Relatives

Red raspberry, a member of the Rosoideae subfamily (Rosaceae), comprises over 740 described species worldwide, with two recognized diploid subspecies: the European *R. idaeus* subsp. *idaeus* and the North American *R. idaeus* subsp. *strigosus* [94,95]. Cultivated raspberries are diploid (2*n* = 2*x* = 14), with domestication involving both subspecies across more than 2000 years of cultivation in Europe and approximately 150 years in North America [96,97]. Evolutionary origins trace to Eurasian forests, with natural distribution extending from Western Europe through Siberia to eastern Asia, and separate colonization of North America during Pleistocene glacial periods [98]. Phylogenetic analysis using chloroplast genome sequences places raspberry within subgenus *Idaeobatus*, with sister relationships to *R. corchorifolius* and close affinity to Korean raspberry (*R. crataegifolius*) [99]. Biogeographic reconstruction indicates Eurasia as the center of diversification.

Wild relatives and inter-subspecific hybrids (*R. idaeus* × *R. strigosus*) are maintained in germplasm banks and serve as crucial donors of antioxidant-related traits, with genomic analyses confirming synteny across the seven chromosomes [95,97,100]. Species diversity within raspberry germplasm includes multiple interfertile taxa. The *R. idaeus* complex comprises the European subspecies *idaeus* (2*n* = 2*x* = 14), a source of high sugar content, and diverse flavor profiles [96], and the North American subspecies *R. strigosus* (2*n* = 2*x* = 14), which contributes cold hardiness, disease resistance, and unique anthocyanin profiles [97,101]. Related species provide specialized traits: *R. occidentalis* (black raspberry, 2*n* = 2*x* = 14) is notable for exceptional anthocyanin content and unique cyanidin-3-rutinoside accumulation [102]. Wild population diversity studies using SSR and SRAP markers reveal significant population structure across natural ranges, with diversity centers in the Caucasus region (European populations) and the Pacific Northwest (North American populations), both serving as priority collection sites [98,103]. Germplasm repositories maintain over 1500 accessions globally, with core collections at USDA Corvallis (~450 accessions), Agriculture Canada (~320 accessions), and major European collections, representing maximum allelic diversity for breeding applications [95].

Commercial raspberry production focuses on antioxidant-rich cultivars such as ‘Haida’, ‘Chilli wack’ and ‘Meeker’, spanning different ripening seasons. European-derived cultivars are reported with several important commercial traits such as high anthocyanin content, excellent antioxidant stability during storage [104], elevated ellagitannin levels and superior ORAC values [105]. Several early-season cultivars also contain high total phenolic content and spine-free canes that facilitate harvest [106]. North American selections include genotypes with cold-hardy characteristics notable for unique anthocyanin glycosylation patterns and high vitamins, as well as others which combine disease resistance with elevated flavonol content and extended shelf-life due to antioxidant stability [104]. Interspecific hybrids (*R. idaeus* × *R. occidentalis*) contribute to genetic diversity, providing genotypes with exceptional anthocyanin profiles (including cyanidin and pelargonidin derivatives) and dual-purpose potential for both fresh consumption and processing [102]. Beyond established cultivars, advanced breeding lines specifically selected for enhanced antioxidant profiles exhibit 40–60% higher total phenolic content than standard cultivars, with specialized metabolite compositions tailored for functional food applications [54,105]. Fruits of several, mainly Polish cultivars of floricane- and primocane-fruiting red raspberry, black raspberry and blackberry, grown in central Europe during two successive vegetation periods, were investigated. The content of phenolic compounds, including anthocyanins, as well as antioxidant properties of fruit extracts were analyzed. Several methods were employed: ferric ion reducing antioxidant power (FRAP), cupric ion reducing antioxidant capacity (CUPRAC), 2,2-diphenyl-1-picrylhydrazyl (DPPH) radical scavenging capacity involving both colorimetric and EPR spectrometric measurements. From among all the tested fruits black raspberries had the largest antioxidant capacity as verified by all methods used in this study. These berries were also the most abundant in phenolic and anthocyanin compounds. Blackberries were characterized by larger antioxidant capacity than red raspberry fruits which had higher content of total phenolics and anthocyanins. Berries of primocane-fruiting cultivars, often used for intensive agricultural production, generally did not differ in the total phenolic and anthocyanin content as well as in the antioxidant capacity as compared to the traditional, floricane-fruiting ones. The research contributes to deep characterization of central European berry fruits which due to their high content and large diversity of health-beneficial compounds are classified as natural functional food [53,106]. Notably, cultivars such as ‘Saanich’, ‘KiwiGold’, and ‘Summit’ have demonstrated superior antioxidant activity and mineral content, with ‘Saanich’ showing exceptional iron and manganese levels and ‘KiwiGold’ ranking among the highest in potassium and phenolics [53]. Additionally, non-commercial cultivars like ‘Adelita’, ‘Glen Dee’, ‘Himbo Top’, ‘San Rafael’, and ‘Tula Magic’ have shown promising antioxidant potential and polyphenol content, suggesting their suitability for future breeding programs targeting nutritional enhancement [107]. Recent evaluations of cultivars grown in northern Mexico further highlight ‘Red Autumn Bliss’ for its high anthocyanin concentration and antioxidant capacity, and ‘Yellow Autumn Bliss’ for its exceptional levels of total phenolics, ellagic acid, ascorbic acid, and polyphenolic summation, reinforcing their value in antioxidant-focused breeding strategies [108].

#### 3.2.2. Modern Genomic and Breeding Tools for Enhancing the Antioxidant Character of the Raspberry

Raspberry antioxidant quality is regulated by constitutive genes that sustain baseline metabolite production during fruit development. Three polyketide synthase (PKS) genes (*PKS1*, *PKS2*, and *PKS3*), have been characterized from raspberry cell cultures, with *RiPKS1* encoding a typical CHS active in naringenin production, *RiPKS3* producing mainly p-coumaroyltriacetic acid lactone (CTAL), *RiPKS2*, an inactive form, and a newly identified, *RiPKS4*, with a crucial role in enhancing ketone levels in raspberry leaves and fruit [109,110]. Another study investigated antioxidant-related gene expression during fruit development in yellow, red, and black raspberries, revealing cultivar- and stage-specific patterns. Genes encoding antioxidant enzymes (SOD, POD, Peroxiredoxin-PRDX, CAT) and flavonoid/anthocyanin biosynthesis enzymes (PAL, FLS, ANR, LAR, UFGT) were found differentially expressed, with expression generally peaking at ripening stages. Transcription factor genes belonging to the basic Helix–Loop–Helix (bHLH), myeloblastosis (MYB), and WRKY families also displayed distinct expression profiles, demonstrating their regulatory role in antioxidant metabolism [111]. Baseline phenolic acid biosynthesis relies on phenylpropanoid pathway genes such as *PAL*, *C4H*, and *4CL*, which have been characterized in raspberries [59,112]. All the above-mentioned studies provide a valuable reference for understanding the molecular basis of antioxidant regulation in raspberry fruit. However, only a limited number of these genes have been functionally annotated and characterized (Table 3), and further research is needed to exploit their roles in enhancing the antioxidant capacity of raspberries.

Genomic resources for raspberries are rapidly expanding and include a chromosome-scale diploid reference genome (~240 Mb, 2*n* = 2*x* = 14) assembled using PacBio long reads and Hi-C scaffolding, comprising 32,831 predicted genes with comprehensive functional annotation [100]. The genome architecture reveals seven linkage groups with high synteny to *Fragaria* and other Rosaceae species. Comparative genomics highlights gene family expansions in secondary metabolite biosynthesis, particularly phenylpropanoid-related genes—as well as disease resistance genes. Prominent tandem gene clusters encode flavonoid biosynthetic enzymes, terpene synthases, and glycosyltransferases. QTL mapping and SNP-based studies have identified loci associated with anthocyanin composition and antioxidant traits [113,114]. A review study by McCallum et al. [115], in raspberry further reported QTLs directly associated with anthocyanin traits, antioxidant capacity, and phenolic content. These include QTLs for anthocyanin composition, such as specific glycosides of cyanidin and pelargonidin, and the mapping of candidate genes underlying anthocyanin/phenolic biosynthesis (structural and regulatory genes/transcription factors such as bHLH, MYB, and basic-leucine zipper transcription factor-bZIP), which often colocalize with QTLs for pigmentation and anthocyanin-related traits. Investigation of gene relative expression as well as transcriptome resources provide datasets related to antioxidant characteristics at diverse developmental stages, tissues, and environmental conditions, supporting functional genomics and breeding applications [100,111].

### 3.3. Blackberry (Rubus occidentalis/Rubus fruticosus *agg*.)

#### 3.3.1. Domestication and Wild Relatives

Blackberry (*Rubus* subgenus *Rubus*) represents a taxonomically and genetically complex crop group characterized by polyploidy, hybridization, and broad ecological adaptation. The cultivation history spans more than 150 years, though systematic breeding only intensified from the 1960s onward [116,117]. Evolutionary origins are geographically diverse: European species such as the *R. fruticosus* aggregate contributed to Old World germplasm, while *R. allegheniensis* and *R. argutus* formed the basis of North American breeding programs [95]. The species exhibit remarkable cytogenetic diversity, with chromosome numbers ranging from diploid (2*x* = 14) to dodecaploid (12*x* = 84), though most commercial cultivars are tetraploid (2*n* = 4*x* = 28) or hexaploid (2*n* = 6*x* = 42) [117]. Domestication has relied heavily on interspecific hybridization between taxonomically distinct lineages, generating highly heterozygous populations and enabling transgressive segregation for traits such as flavor, yield, and antioxidant content [116]. Genomic resources now support the study of this complexity, with *R. occidentalis* (black raspberry, ~290 Mb) sequenced using PacBio and Hi-C technologies [100], while the *R. argutus* ‘Hillquist’ genome (~270–298 Mb) offers a high-quality chromosome-scale reference for dissecting antioxidant-related traits in polyploid backgrounds [97].

Wild *Rubus* relatives remain an essential reservoir of antioxidant and adaptive diversity. The *R. fruticosus* aggregate, comprising more than 400 microspecies (agamospecies) maintained through apomixis, provides stable sources of anthocyanins, ellagitannins, and pathogen resistance [95]. European populations exhibit anthocyanin concentrations as high as 589 mg/100 g FW and display unique ellagitannin profiles; however, this value pertains to specific *R. fruticosus* accessions used in food colorant studies and does not necessarily represent wild populations in general [102]. North American species contribute complementary adaptive traits: *R. allegheniensis* combines cold hardiness and unique terpene profiles; *R. argutus* contributes heat tolerance, extended harvest windows, and high phenolic diversity; and *R. ursinus*, the Pacific dewberry, imparts exceptional flavor and rare anthocyanin glycosides [118]. Further genetic breadth derives from Mexican wild species with tropical adaptation and continuous fruiting potential, though direct citation is limited and may be supported by regional germplasm surveys or USDA reports. Asian taxa such as *R. corchorifolius* and *R. phoenicolasius* contribute disease resistance, stress tolerance, and novel bioactive compounds [119]. Comparative studies using AFLP markers reveal that North American blackberry cultivars have a narrower genetic base compared with European wild populations, underscoring the need to broaden germplasm pools in breeding programs [120]. Conservation initiatives now safeguard over 800 accessions globally, capturing diverse lineages to ensure sustainable exploitation of antioxidant-related traits [121].

Commercial blackberry cultivars display marked variation in antioxidant composition, reflecting their diverse pedigrees. Erect thorny selections have been reported with high total phenolic content and anthocyanin levels, showing excellent ORAC values and storage stability [122]. Thornless erect cultivars display high ellagitannin accumulation, malvidin-rich anthocyanin profiles, and shelf-life extension, while semi-erect genotypes achieve total antioxidant capacities up to 11,200 μmol TE/100 g, with consistent phenolic retention under stress [105]. Trailing cultivars from the Pacific Northwest dominate the processing and fresh markets due to their distinctive flavor, anthocyanin diversity, and superior postharvest vitamin C retention [104,117]. Recent metabolomic and pharmacological profiling identified several high-antioxidant cultivars such as ‘Kiowa’, which exhibits the highest antioxidant and anti-inflammatory activity, driven by high levels of quercitrin, luteolin, and cyanidin 3-O-glucoside. Cultivar ‘10-5n-2’ contains strong total anthocyanin and polyphenol content, while ‘Chester’ is noted for strong fruit firmness and high antioxidant retention; and ‘Shuofeng’ combines high yield with excellent antioxidant capacity. Additional cultivars such as ‘Thorn Free’, have been reported with notably high cyanidin-3-glucoside and total phenols concentrations as well as ‘Loch Ness’, which exhibits very high total phenols and strong antioxidant capacity, further exemplify the nutritional potential of thornless selections [123], while ‘Triple Crown’ and ‘Encore’ exhibit exceptionally high antioxidant activity, with DPPH values of 184.43 and 316.02 µg/mL, respectively, and strong FRAP values, highlighting their potential for breeding antioxidant-rich genotypes [53]. Complementing cultivated germplasm, wild relatives such as *R. ulmifolius*, *R. perrobustus*, *R. wimmerianus*, *R. pedemontanus*, and *R. grabowskii* also display exceptionally high phenolic content in fruits and leaves, offering unique opportunities for bioactive compound enrichment in breeding [124].

#### 3.3.2. Modern Genomic and Breeding Tools for Enhancing the Antioxidant Character of the Blackberry

Blackberry fruit quality depends on constitutive antioxidant production systems that operate throughout normal fruit development. In a study by Huang et al. [125], the authors investigated the molecular basis of antioxidant accumulation during fruit ripening in blackberries by monitoring the expression of structural and defense-related genes. They collected blackberry fruits at different developmental stages (green, red, and fully black) and quantified the gene expression through transcriptome profiling combined with quantitative RT-PCR. They reported that genes such as *PAL*, *CHS*, *CHI*, and *F3H* exhibited higher expression at early developmental stages, establishing the metabolic framework for downstream flavonoid biosynthesis. In contrast, the late anthocyanin biosynthetic genes, namely *DFR*, *ANS*, and *UFGT*, showed pronounced up-regulation during the red-to-black transition, coinciding with the rapid accumulation of anthocyanins. Antioxidant defense enzymes including SOD, CAT, APX, and POD, were significantly induced at the late ripening stage, reflecting their role in mitigating oxidative stress during fruit maturation. Table 4 presents the genes that have been functionally annotated and characterized until today according to the international literature findings.

Genomic resources for blackberries are rapidly advancing, with recent efforts focused on developing chromosome-scale reference genomes for diploid *R. argutus* (‘Hillquist’, ~298 Mb) and tetraploid BL1 (~919 Mb), assembled using PacBio, Oxford Nanopore, and Hi-C technologies [132,133]. These assemblies reveal complex subgenome relationships shaped by ancient polyploidization and diploidization, with gene family expansions in secondary metabolite biosynthesis, particularly phenylpropanoid and flavonoid pathways, and stress-response genes. Transcriptomic datasets across fruit developmental stages and tissue types support functional annotation and breeding applications [134]. Resequencing of seven tetraploid cultivars uncovered candidate genes for antioxidant traits and fruiting habits [133]. Early diversity studies using AFLP markers laid the foundation for pangenome initiatives, suggesting an accessory genome comprising 15–20% of total genes enriched for secondary metabolism and environmental response [135]. Likewise, Chizk et al. [60], performed a large-scale GWAS on 300 tetraploid blackberry genotypes to identify genetic factors controlling fruit firmness and red drupelet reversion (RDR). Their findings demonstrated that postharvest quality traits are moderately heritable and influenced by multiple small-effect QTLs, including loci linked to polygalacturonase, pectin methylesterase, and glucanase related genes. Overall, the research highlighted that postharvest quality is genetically complex but provided candidate loci and datasets for future genomic selection.

### 3.4. Cranberry (Vaccinium macrocarpon *Ait*.)

#### 3.4.1. Domestication and Wild Relatives

American cranberry is a diploid species (2*n* = 2*x* = 24) native to acidic bog ecosystems of North America, cultivated primarily in the United States, Canada, and Chile for its high polyphenol content and associated health benefits. It represents one of only three widely cultivated fruit crops native to North America, with domestication dating back approximately 200 years ago [54,127]. Natural populations span acidic wetlands from Newfoundland to North Carolina and west to Minnesota, with genetic diversity centers in Wisconsin, Massachusetts, and New Jersey bog regions [136]. Phylogenetic analyses using chloroplast markers indicate that *V. macrocarpon* diverged from other cranberry species 5–10 million years ago, followed by population expansion after Pleistocene glacial retreats [137,138]. Early domestication involved selection from wild bog populations, showing minimal genetic bottlenecks compared to other crops. Modern breeding began in the 1950s, initially targeting fruit quality and yield, and later expanded to include enhanced antioxidant content and anthocyanin profiles, as confirmed by genotyping and metabolomic studies of breeding cycles [139,140].

Cranberry genetic resources include both wild populations and related species. Wisconsin bog populations harbor the largest genetic diversity, with over 150 distinct genotypes serving as sources of disease resistance and unique antioxidant profiles [136,141]. Massachusetts populations provide cold hardiness traits and elevated proanthocyanidin content [142], while New Jersey Pine Barrens populations contribute heat tolerance, unique A-type proanthocyanidin variants, and extended harvest characteristics [66]. Canadian populations exhibit extreme cold tolerance, specialized flavonoid profiles, and late season ripening traits [143]. Related species, including *V. oxycoccos* (small cranberry) and *V. microcarpum*, are genetically distinct wild relatives of *V. macrocarpon*. They contribute specialized traits such as cold hardiness, disease resistance, high anthocyanin content, unique glycosylation patterns, and stress tolerance [66]. Microsatellite marker analyses of wild cranberry (*V. macrocarpon*) clones from Newfoundland and Labrador revealed significant population structure correlated with geography, along with high allelic richness and diverse antioxidant properties, highlighting these Canadian populations as priority resources for cranberry conservation and breeding [143]. The USDA’s National Clonal Germplasm Repository (NCGR) maintains over 300 cranberries and cranberry-wild relative accessions (including ~146 *V. macrocarpon*) in its *Vaccinium* collection. Genetic studies of cultivars and accessions have identified dozens of unique genotypes among these accessions for example, 64 unique genotypes out of 271 plants in 77 accessions confirming substantial genotypic diversity in the repository [144]. Recent chromosome-level genome assemblies for *V. macrocarpon* and *V. microcarpum* further provide foundational resources for trait and antioxidant biosynthesis analyses [141].

Commercial cranberry cultivars are selected for enhanced antioxidant profiles across early, mid, and late harvest seasons. Early-season cultivars are noted for high anthocyanin and total phenolic content, excellent processing quality, elevated proanthocyanidin levels, and superior antioxidant stability [142]. Heritage cultivars such as ‘Early Black’ and ‘Howes’ also fall into this category, showing strong antioxidant capacity and minimal genetic bottlenecks, with ‘Howes’ exhibiting exceptionally high anthocyanin concentrations and ‘Early Black’ known for stable phenolic content across ripening stages [145]. Mid-season cultivars such as ‘Stevens’ serve as industry standards with balanced antioxidant profiles and high A-type proanthocyanidin content, while others like ‘Ben Lear’ exhibit elevated total phenolics, unique anthocyanin glycosylation patterns, and strong cold hardiness. ‘McFarlin’, another heritage cultivar, contributes moderate antioxidant levels and maintains high genetic diversity, making it valuable for breeding and conservation. Late-season cultivars are recorded with exceptional anthocyanin concentrations and the highest observed ORAC values, with selections like ‘Bergman’ noted for elevated flavonol content and superior antioxidant retention during processing [146]. Additional high-antioxidant cultivars include ‘Crimson Queen’, known for its high anthocyanin and proanthocyanidin content with excellent color stability; ‘Mullica Queen’, which demonstrates elevated total phenolics and strong antioxidant retention during processing; ‘Scarlet Knight’, rich in flavonols and anthocyanins and bred for functional food applications; ‘HyRed’, valued for its deep red pigmentation and high ORAC values; and ‘Demoranville’, which combines high proanthocyanidin levels with improved disease resistance [140]. Specialized selections provide deep red pigmentation, diverse anthocyanin profiles, enhanced proanthocyanidin content, and improved disease resistance, making them ideal for premium fresh markets and functional food applications [145]. Finaly, ‘Kalnciema Agrā’ and ‘Kalnciema Tumšā’ exhibit high proanthocyanidin and triterpenoid content, contributing to strong antioxidant activity. ‘Searles’ is notable for elevated proanthocyanidins, while ‘Black Veil’ stands out with high flavonol levels. ‘Red Star’ combines high chlorogenic acid with triterpenoids, and ‘Pilgrim’, despite lower anthocyanins, shows significant antioxidant capacity due to its triterpenoid content [145,147].

#### 3.4.2. Modern Genomic and Breeding Tools for Enhancing the Antioxidant Character of the Cranberry

Cranberries are rich in phenolic compounds, particularly flavonoids and proanthocyanidins, which have been widely studied for their diverse biological activities [148]. Breeding strategies have primarily focused on traits prioritized by the industry, such as fruit quality characteristics, including firmness, size, and anthocyanin content, as well as resistance to fruit rot, which are considered the most desirable attributes in new cranberry cultivar releases [149]. Several studies suggest that advanced DNA-based selection strategies could significantly benefit the global cranberry industry [149], while others emphasize that integrating genetic diversity analysis with antioxidant profiling using DNA markers (ISSR, EST-SSR, EST-PCR) is crucial for conserving wild cranberry germplasm and identifying diverse, antioxidant-rich genotypes for future breeding and crop improvement [143]. Vorsa and Zalapa [56], reviewed the biology, domestication history, key traits, and breeding challenges of American cranberry, highlighting recent molecular advances and proposing marker-assisted selection (MAS), as a crucial tool for future genetic improvement. They noted that the QTLs identified for important traits like productivity, berry size and anthocyanins content pointing to the value of molecular tools and MAS to overcome traditional breeding limitations and accelerate genetic improvement. Debnath et al. [150], used DNA-based markers, particularly ISSRs, to analyze genetic diversity in wild and cultivated berry crops, showing that these markers effectively differentiated genotypes and are useful for cultivar identification and breeding, while also reporting wide variation in antioxidant activity that did not align with molecular diversity patterns, highlighting the need to combine both approaches for germplasm conservation and crop improvement. Recently Jiménez et al. [151], analyzed phenotypic and genetic variation for eight flavonol compounds in a genetically diverse germplasm collection to evaluate the application of genomic prediction in breeding programs. By performing flavonol quantification and genome wide association studies they estimated breeding values (GEBVs) for total flavonol content. They concluded that although further investigation is needed to improve prediction accuracy, simulated crosses showed similar outcomes between phenotypic and genomic selection, showing the potential of genomic tools to enhance flavonol content and fruit quality in cranberry breeding programs.

A recent genome-wide association study of 362 cranberry accessions, including wild germplasm and first-, second-, and third-generation breeding cycles, revealed that early breeding efforts were targeted towards increased fruit weight, yield, and anthocyanin content, while other fruit quality parameters, such as Brix and acidity, remained relatively stable. Marker–trait associations identified for fruit weight and fruit rot resistance represent important genomic resources that can be exploited to accelerate molecular breeding in *Vaccinium* crops [139]. However, breeding efforts specifically targeting fruit antioxidant improvement remain largely unexplored in cranberries. Moreover, while genetic tools such as CRISPR/Cas9 have begun to be applied in other berry species like blueberries [144,145], these efforts have primarily focused on traits unrelated to antioxidant capacity. To date, no reports exist on the application of such genome-editing tools in cranberries.

### 3.5. Grapevine (Vitis vinifera *L*.)

#### 3.5.1. Domestication and Wild Relatives

Grapevines are among the most economically important crops cultivated worldwide. Global vineyard coverage spans about 7.2 million hectares, producing 77.8 million tons of grapes annually of which 47.4% is used for winemaking, 44.5% for fresh consumption, and 8% for dried grapes (OIV). Grapes are classified into two subgenera: *Muscadinia*, (2*n* = 40) and the larger subgenus *Euvitis* (including *V. vinifera*), with a basic chromosome number of 2*n* = 38. Grapevine domestication dates back approximately 8000–11,000 years and archaeological and genetic evidence points to Western Asia (a region between the Black Sea and Iran) as the primary center of origin, though multiple domestication centers have also been proposed [152,153,154]. The *Vitis* genus comprises 60–80 species characterized by exceptionally high genetic diversity. Ampelographic and genetic analyses suggest that between 6000 and 10,000 *V. vinifera* cultivars are currently cultivated worldwide [155]. Global germplasm inventory repositories, such as the Vitis International Variety Catalogue (VIVC), have catalogued thousands of cultivars and wild accessions, providing valuable resources for elucidating phylogenetic relationships and trait variation.

Wild *Vitis* relatives exhibit important traits not found in *V. vinifera* that have been associated mainly with resistance/tolerance to biotic/abiotic stresses [156]. Historically, European *V. vinifera* grapevines were grafted onto American varieties derived from wild *Vitis* species to combat the devastating phylloxera epidemic (*Daktulosphaira vitifoliae*) [157]. Moreover, over the years, breeding efforts utilizing crosses of *V. vinifera* with wild species such as *V. arizonica* and *Muscadinia rotundifolia* [Michx.] Small (2*n* = 40), managed to develop lines with resistance to devastating bacterial and fungal diseases like ‘Pierce’s disease-PD’, downy mildew (*Plasmopara viticola*) and (powdery mildew (*Erysiphe necator*), respectively [158].

In the context of antioxidant capacity, the wild relative *M. rotundifolia*, a grape species native to the southeastern United States, has drawn much interest recently from the food, pharmaceutical, and nutraceutical industries owing to their unique chemical composition and nutritional value [159]. Muscadine grapes are rich in distinctive primary and secondary metabolites, including notable phenolics like gallic acid, ellagic acid, proanthocyanidins, catechins, quercetin, resveratrol, and myricetin. Muscadine-derived bioactive compounds exhibit strong antioxidant, anticancer, antimutagenic, antimicrobial, and anti-inflammatory properties, and thus hold tremendous health promoting potential [160]. They are also essential contributors to grape quality traits and their wide flavor diversity.

#### 3.5.2. Modern Genomic and Breeding Tools for Enhancing the Antioxidant Character of Grapevine

The grapevine was among the first fruit crops to be sequenced, with the reference genome of the near-homozygous Pinot Noir PN40024 line published by Jaillon et al. [161]. The diploid genome is made up of 19 chromosomes and covers about 487 Mb. The assembly has been improved to chromosome-scale resolution by further updates that included BAC-by-BAC sequencing, Illumina short reads, and long-read data [162]. Gene families engaged in secondary metabolism were revealed by annotation, particularly those associated with the biosynthesis of terpene and polyphenol compounds, which include antioxidants like anthocyanins and stilbenes (e.g., resveratrol) [163]. These genomic resources have allowed identification of key genes such as *VvDXS1*, *VvbZIP61* (terpene synthesis) *VvCHS*, *VvMYBA1* and *VvSTS* (polyphenol synthesis) facilitating marker-assisted breeding and functional genomics in grapevines. Table 5 presents the genes that have been functionally annotated and characterized regarding their association with antioxidant levels.

Advances in DNA sequencing technologies have yielded enhanced grapevine reference genome assemblies [164] and a non-reference pangenome derived from wild grapevine accessions [165]. These genomic resources provide powerful tools for elucidating the molecular basis of key morphological, physiological, and biochemical traits that influence yield, fruit quality, and stress tolerance. Notably, the recent development of a comprehensive grapevine pangenome reference map built from resequencing data of 466 cultivars and capturing an extensive array of both short variants and structural variants (SVs), is expected to shed light on complex polygenic trait associations and accelerate genomic-assisted breeding efforts [166].

Transcriptomics and metabolomics are increasingly employed to investigate molecular processes in grapevines and have been focusing either on plant and fruit development or on interactions with abiotic and biotic factors. Integrating these approaches has become critically important for understanding key physiological and metabolic pathways [159]. Insights gained from such analyses can be directly applied in breeding programs, using genes linked to improved fruit quality, such as those enhancing the accumulation of health-promoting compounds like antioxidants, as molecular markers for crop improvement.

Over the past two decades, a wealth of transcriptome and metabolome data has been produced which greatly aid the investigation of grapevine berry physiology, secondary metabolism, and quality traits [167,168,169,170,171]. Integrated omics datasets can be compiled into user-friendly resources for the research community, enabling exploration of potential gene–gene and gene–metabolite interactions that can later be validated through hypothesis-driven experiments. One such resource is the Grapevine Transcriptomic and Metabolomic Integrated Database (TransMetaDb), available through the Vitis Visualization (VitViz) platform (https://tomsbiolab.com/vitviz accessed on 30th August 2025) which provides a valuable tool for linking antioxidant potential with berry development and metabolism [171].

**Table 5:** Genes analyzed for their functional involvement in the antioxidant capacity of grapevine.

Enzyme Class	Gene Name	Functional Role/Association with Antioxidant Levels	References
Basic-leucine zipper transcription factor	*VvbZIP61*	Monoterpene metabolism Associated with increased levels of monoterpenes	Zhang et al., 2023 [172]
1-deoxy-d-xylulose-5-phosphate synthase (DXS)	*VvDXS1*	Rate-limiting enzyme of the MEP pathway; Major determinant of monoterpenoid content	Yang et al., 2017 [173] Zhang et al., 2025 [174]
Isopentenyl pyrophosphate synthases (IPPS) (geranylgeranyl pyrophosphate synthase)	*VvGGPPS-LSU*	Enzyme of the MEP terpene biosynthesis pathway; Associated with increased accumulation of monoterpenoid and norisoprenoid levels.	Zhang et al., 2025 [174]
WRKY transcription factors	*VvWRKY24*	A key regulator of isoprenoid metabolism. Associated with enhanced levels of β-damascenone, an isoprenoid important for berry and wine aroma	Wei et al., 2025 [175]
MYB transcription factors	*VvMYBA1*	A key positive regulator of anthocyanin biosynthesis	Liu et al., 2023 [65]
Chalcone synthase (CHS)	*VvCH2*	A key enzyme in the phenylpropanoid metabolism committed to the synthesis of flavonoids	Lai et al., 2025 [176]
Stilbene synthases (STS)	*VvSTS*	A key enzyme of the phenylpropanoid pathway committed to the synthesis of resveratrol	Lai et al., 2025 [176]

In grapevines, antioxidants comprise large classes of compounds, including secondary metabolites involved in shaping the aroma and flavor of grapes and wine (e.g., terpenoids), compounds related to health benefits (e.g., polyphenols, resveratrol, anthocyanins), and compounds determining the responses to biotic and abiotic stresses (e.g., stilbenes, anthocyanins, terpenes) [177]. Despite the importance of antioxidant content as a key quality trait in grapevine breeding, the genetic basis underlying the biosynthesis and accumulation of aroma/flavor-related terpenoid compounds or phenolic, flavonoid and anthocyanin molecules remains obscure due to the complexity of the respective metabolic pathways that involve a multitude of enzymes and gene networks [178]. Moreover, reliable molecular markers linked to these traits are largely lacking, posing a challenge for the implementation of MAS in antioxidant-enhancement breeding and pointing to the necessity of adopting modern genomic tools and molecular breeding strategies [179].

##### Antioxidants Related to the Aromatic Profile of Grapes and Wine

**Figure 2:**
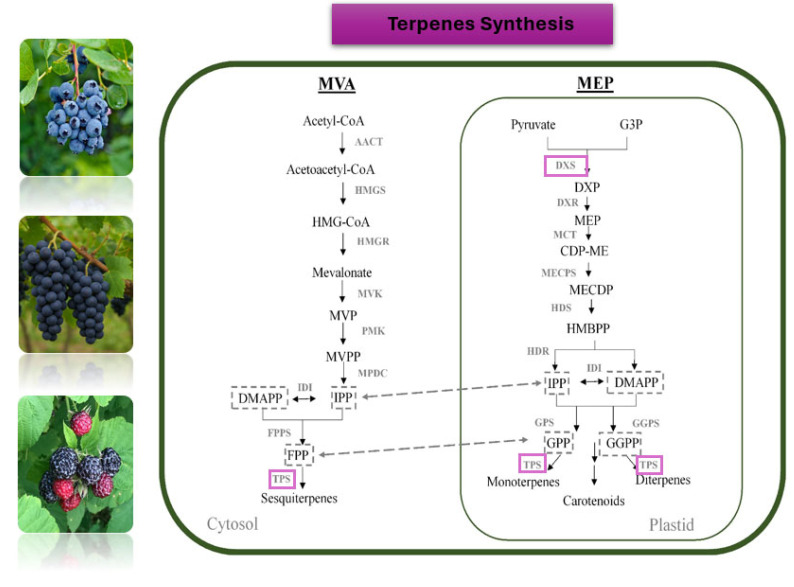
A general model of the mevalonate (MVA) and methylerythritol phosphate (MEP) pathways, operating in the cytosol and plastid, respectively, generate the prenyldiphosphate precursors required for terpene biosynthesis; downstream metabolic products and respective enzymes are indicated. AACT, acetyl-CoA acetyltransferase; CDP-ME, 4-diphosphocytidyl-2-C-methyl-D-erythritol; CDP-MEP, CDPME 2-phosphate; CMK, 4-(cytidine 5′-diphospho)-2-C-methyl-D-erythritol kinase; DMAPP, dimethyl allyl diphosphate; DXP, 1-deoxy-D-xylulose 5-phosphate; DXS, DXP synthase; DXR, 1-deoxy-D-xylulose 5-phosphate reductoisomerase; FPP, farnesyl diphosphate; FPPS, FPP synthase; G3P, glyceraldehyde 3-phosphate; GGPP, geranylgeranyl diphosphate; GGPPS, GGPP synthase; GPP, geranyl diphosphate; GPPS, GPP synthase; HDR, hydroxymethylbutenyl diphosphate reductase; HDS, 4-hydroxy-3-methylbut-2-en-1-yl diphosphate synthase; HMBPP, (*E*)-4-hydroxy-3-methylbut-2-en-1-yl diphosphate; HMG-CoA, hydroxymethylglutaryl-CoA; HMGR, HMG-CoA reductase; HMGS, HMG-CoA synthase; IDI, isopentenyl pyrophosphate isomerase; IPP, isopentenyl diphosphate; MCT, 2-C-methyl-D-erythritol 4-phosphate cytidylyltransferase; MECPD, 2-C-methyl-D-erythritol 2,4-cyclodiphosphate; MECPS, MECPD synthase; MVK, mevalonate kinase; MPDC, mevalonate diphosphate decarboxylase; MVP, mevalonate 5-phosphate; MVPP, mevalonate 5- pyrophosphate; PMK, phosphomevalonate kinase; TPS, terpene synthase (modified from Bosman et al., 2023 [183]).

The complex bouquet of grape aroma is attributed to a diverse array of volatile organic compounds, such as benzenoids, esters, norisoprenoids, and terpenoids [180,181]. Distinct classes of terpenoids like mono- and sesquiterpenes contribute significantly to the characteristic aromatic profiles of individual grapevine cultivars. For example, monoterpenoid alcohols such as linalool, nerol, and geraniol are key contributors to the floral and citrus-like aromas typical of highly aromatic varieties such as ‘Muscat’ and ‘Gewürztraminer’. On the other hand, sesquiterpenes, such as rotundone, are associated with spicy, peppery notes, particularly characteristic of the ‘Syrah’ cultivar [182]. Despite numerous investigations, thus far, the genetic and regulatory aspects of terpenoid production have not been fully uncovered due to the complexity of the particular biosynthetic pathway. Monoterpenoids and sesquiterpenes are synthesized via the mevalonate pathway (MVA) and the methylerythritol phosphate (MEP) pathway which are compartmentalized into the cytoplasm and plastids, respectively (Figure 2). The 1-deoxy-d-xylulose-5-phosphate synthase (DXS) and HMG-CoA reductase (HMGR) are the major contributors to metabolic flux control of the pathways, respectively whereas terpene synthases (TS) functioning in the final steps are associated with the synthesis of distinct terpenoid molecules from prenyldiphosphate precursors [183]. A large family of terpene synthases (TS) encoded by 69 genes identified in the grapevine genome is considered to play critical roles in terpenoid biosynthesis [177]. Structurally diverse terpene synthases acting on prenyldiphosphate precursors are considered to drive the wide range of terpenes produced in grapes. These terpenes can then undergo secondary modifications, such as glycosylation and oxidation, further expanding the diversity of grape and wine aromas and flavors [183]. In addition, transcription factors such as members of the NAC, MYB, and AP2/ERF families, have been suggested to play roles in the transcriptional regulation of terpene synthesis genes in grapevine [184]. However, the functional characterization of most TS genes/enzymes or transcription factor genes has been limited. Similarly, the oxidation, reduction, and glycosylation processes that lead to the production of monoterpenoid derivatives -also key aroma compounds- are poorly understood [172].

Early QTL mapping and GWAS demonstrated that a major QTL linked to aroma profiling and monoterpenoid content was associated with the *VvDXS1* gene. A series of causal SNPs were identified mainly in the coding region of the gene affecting the catalytic activity of the DXS1 enzyme, as well as in the regulatory region affecting gene expression levels [173,185,186,187,188]. Thus, *DXS1* was proposed as an effective molecular marker for an aroma-related quality trait; nevertheless, various aromatic varieties and their aroma/flavor variabilities were found unrelated to *DXS1,* highlighting the need for further gene mining and development of additional genetic markers.

An integrative approach combining QTL mapping, transcriptomic analysis, and callus transformation identified additional genes associated with monoterpenoid composition and abundance. The F_1_ progeny derived from a cross between an aromatic cultivar (‘Beifeng’) and a neutral cultivar (‘3–34’) revealed stable QTLs associated with eleven monoterpenes. Parallel RNA-seq analysis of thirty-four grapevine cultivars with distinct aromatic profiles demonstrated a significant correlation between the expression of genes located within these QTL regions and the monoterpenoid content of berries. Among these, *VvbZIP61*, encoding a basic leucine zipper (bZIP) transcription factor involved in multiple plant processes, was highlighted as a key regulator. Functional validation through *V. amurensis* callus overexpression confirmed its role in monoterpenoid metabolism, leading to a significant increase in the accumulation of several monoterpene compounds [172].

To identify additional candidate genes associated with monoterpenoid levels, Zhang et al. [174], employed GWAS on an F1 population derived from crosses between a typical muscat variety (‘Muscat of Alexandria’) and a nonaromatic variety (‘Christmas Rose’). A range of significant SNPs were identified, all located on chromosome 5, forming a monoterpenoid-related marker cluster. Twenty-five lead SNPs appearing stable over a two-year period were validated in a germplasm population of 97 diverse *Vitis* species and were found highly associated with monoterpenoid levels. The underlying genes encode the DXS enzyme, a glycoside hydrolase, an ABC transporter, and a geranylgeranyl pyrophosphate synthase-large subunit (VvGGPPS-LSU) involved in the terpene synthesis pathway. Notably, the functionality of the latter in increasing monoterpenoid levels was verified in vivo through transformation and overexpression in transgenic calli, reinforcing the reliability of the marker cluster. Importantly, the SNP markers were converted to a KASP marker set, enabling direct molecular breeding applications. 

Besides terpenoid compounds, norisoprenoids, which are derived from the oxidative cleavage of carotenoids, constitute another class of volatile aroma compounds with major contribution to the floral and fruity notes of grape berries and wine. Among them, β-damascenone is the most abundant norisoprenoid found in grapevine [189]. A recent study reported that a WRKY transcription factor, ViWRKY24, plays a role as a key regulator of β-damascenone accumulation in grape berries. Functional characterization through overexpression and gene silencing in grape leaves, berries, and calli revealed that VviWRKY24 modulates norisoprenoid metabolic flux and alters norisoprenoid composition, specifically enhancing β-damascenone levels [175].

The studies described above will further illuminate the regulation of terpenoid and isoprenoid metabolic pathways and facilitate grapevine improvement as it pertains to antioxidant- related aroma/flavor traits.

##### Antioxidants Related to Phenolic Compounds and Health Benefits

In the context of the rising interest in nutraceuticals and phenolic antioxidants found in the skin, flesh, and seeds of grapevine berries, Muscadine grapes, have been gaining much attention in recent years, since they have distinctive flavor and aroma properties and are a major source of essential oils, vitamins, minerals, and antioxidants. Notably, they accumulate significantly higher levels of bioactive polyphenolic compounds compared to other grape species [190,191], enhancing their value both nutritionally and commercially.

Antioxidant activity is higher in skin than pulp and increasing the antioxidant capacity of muscadine berry skin has been an important breeding target. Although many studies have reported on the phenolic content and antioxidant activities in muscadine berries [159], the genetic basis of varied antioxidant profiles has not been studied until recently. Transcriptomic analysis of two contrasting muscadine genotypes differing in phenolic content and antioxidant capacity revealed distinct transcript profiles across berry development that was accompanied by differential total phenolic/total flavonoid contents and antioxidant activities [192]. Differential gene expression was witnessed predominately at the ‘veraison’ stage and involved genes coding for key enzymes in the polyphenol biosynthetic pathway which were found significantly upregulated in the C5 genotype pointing to genotype-specific regulation. On the other hand, genes of the non-flavonoid pathway, stilbene synthases *(STS1*, *STS2*, *STS3)*,exhibited higher expression levels at the ‘veraison’ and ripe berry stages in the C6 genotype, indicating, enhanced, genotype-specific production of stilbenoid compounds in the late berry developmental stages [192]. Similarly, RNA-seq was employed to perform comparative transcriptomic analysis of phenolic pathway associated genes in two muscadine grape varieties, ‘Paulk’ and ‘Supreme’, during berry development. Differential gene expression was observed for *VvPAL* and *VvCHS* which manifested in a genotype- and tissue-specific manner. Conversely, *VvMYBA1* and *VvMYBCS1* were significantly upregulated in ‘veraison’ and mature grape berries in both varieties, suggesting a positive contribution to anthocyanin accumulation [187]. These findings underscore the importance of specific metabolic genes and transcriptional regulators in shaping antioxidant biosynthesis, providing valuable targets for breeding programs aimed at enhancing antioxidant capacity and fruit quality.

To elucidate the genetic architecture of antioxidant quantitative traits in Muscadinia grape, a multi-locus GWAS analysis was conducted to identify genomic loci associated with total phenolic and flavonoid content and the quantitative property of their antioxidant activity [179]. Three hundred fifty genotypes were analyzed through a combination of phenotypic (phenolic content determination and antioxidant activity), berry skin transcriptomic, and GWAS analyses. Twelve Quantitative Trait Nucleotides (QTNs) were significantly correlated with four antioxidant-related traits, and candidate genes were found to be positively and negatively associated with antioxidant activities. Among the positively correlated candidate genes, a *UDP-glycosyltransferase* was identified as a strong candidate significantly correlating with high antioxidant values. UDP-glycosyltransferases are known to be involved in flavonoid and anthocyanin biosynthesis, indicating that the muscadine gene identified in this study may play an important role in enhanced antioxidant capacity. Notably, the gene coding for 4-hydroxy-4-methyl-2-oxoglutarate aldolase was negatively associated with antioxidant values. This gene is involved in the degradation pathway of gallic acid, a key phenolic compound, in bacteria [193], suggesting that it may act as a negative regulator of phenolic antioxidant activity in muscadine grapes. In another study, the role of the VvMYBA1 transcription factor in anthocyanin biosynthesis was further elucidated using a combination of CRISPR/Cas9-mediated knockout of *VvBBX44*, a gene encoding a repressor of *VvMYBA1*, and transient transformation of ‘Jingxiu’ grapevine berries. Loss of *VvBBX44* function led to increased anthocyanin accumulation, whereas overexpression of *VvMYBA1* increased *VvBBX44* expression [65]. These results demonstrate that *VvMYBA1* and *VvBBX44* act together in a regulatory feedback loop to fine-tune anthocyanin biosynthesis, maintaining anthocyanin levels in the desired range.

**Figure 3:**
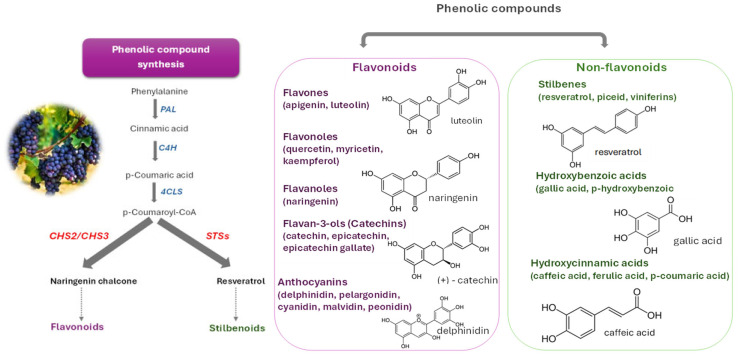
A general model of the phenylpropanoid pathway; Targeted genome editing of *chalcone synthase* (*CHS*) using CRISPR/Cas9 reduces flux into the flavonoid branch of the phenylpropanoid pathway. This shift enhances substrate availability for stilbene synthase (STS), thereby promoting resveratrol biosynthesis and accumulation in transgenic cells. Both flavonoids and stilbenoids originate from the same precursor, p-coumaroyl-CoA, highlighting the competitive relationship between the two pathways.

Along these lines, a notable recent study has reported on the use of targeted editing through CRISPR/Cas9 technology to induce a metabolic shift from the flavonoid to the stilbenoid pathway, thereby promoting resveratrol biosynthesis and accumulation in *V. davidii* transgenic cells [176]. This approach leveraged the fact that both resveratrol and flavonoids are derived from the phenylpropanoid biosynthesis pathway, where stilbene synthase (STS) and CHS compete for the same substrate to produce stilbenoids and flavonoids, respectively (Figure 3). CRISPR/Cas9-based targeted editing of the *VdCH2* gene resulted in efficient CHS2 knock-out leading to suppression of the flavonoid pathway and reduction in flavonoid levels, but to a significant increase in stilbenoid compounds such as resveratrol and its derivatives. These findings offer novel strategies for enhancing targeted antioxidant content in grapevine through redirecting metabolic flux from the flavonoid to the stilbenoid pathway and present an effective alternative to the challenging and costly extraction of resveratrol, a highly valuable antioxidant with beneficial antioxidant properties [176,194].

The findings above set the basis for a deeper understanding of polyphenol accumulation and antioxidant activity and pave the way for enhancing the nutraceutical value of berries through modern breeding and metabolic engineering approaches.

## 4. Challenges and Future Prospects in Berry Breeding for Enhanced Antioxidant Content

Growing consumer awareness of the health benefits of berry consumption has shifted breeding priorities toward improving nutritional quality. Achieving this goal requires the integration of genomic information with phenotypic evaluation to identify genotypes with high antioxidant potential. However, several key challenges limit this progress.

Bioactive compounds such as antioxidants are genetically complex and their expression is strongly influenced by environmental conditions and cultivation practices. Antioxidant accumulation shows high plasticity, due to genotype by environment interactions, temperature, and light exposure, leading to large fluctuations in polyphenol and anthocyanin content across environments [195,196]. This genotype by-environment interaction complicates the establishment of reliable genomic markers for selection. Moreover, polyploidy and high heterozygosity hinder allele dosage estimation and genomic prediction in many berry crops [71,197]. Although high-quality genome assemblies are available for several berry species, the specific roles and functional characterization of many genes involved in antioxidant biosynthesis remain underexplored.

Phenotyping protocols for antioxidant traits are often laborious, destructive, and costly [197]. Emerging imaging and sensor-based methods, such as hyperspectral or computer-vision platforms, offer scalable alternatives but generate complex data not yet fully integrated into genomic prediction pipelines [198,199]. These limitations hinder high-throughput screening across large breeding populations, reducing the feasibility of associating phenotypic data with genomic profiles at an early stage of selection. Moreover, flavor and nutraceutical attributes are not key selection criteria during the initial stages of breeding programs [200], while the limited representation of diverse genetic resources, including wild germplasm, in association studies restricts the discovery of novel alleles responsible for enhanced antioxidant biosynthesis.

Climate change intensifies these challenges. Warmer winters, heatwaves, and irregular precipitation increasingly disrupt chill accumulation, phenology, and fruit quality. In blueberries, reduced winter chilling and elevated temperatures advance flowering and fruiting, leading to uneven ripening and lower quality. Genetic analyses have identified loci linked to low-chill adaptation [200], while observations in Mediterranean-type environments further reveal altered reproductive timing and increased drought sensitivity [201,202]. Raspberries exhibit disrupted dormancy and altered budbreak, affecting fruit size, composition, and flavor [201,202,203], while in blackberries cold storage is used to compensate for insufficient chilling [204,205]. In cranberries, warming advances flowering and heightens frost risk [206]. Overall, these fluctuating environmental stressors disrupt antioxidant production pathways, leading to inconsistent fruit nutritional quality across all berry crops [196,207,208].

The sustainable improvement of antioxidant traits will depend on translating complex omics data into practical breeding decisions, managing polygenic traits under variable climates, and expanding high-throughput phenotyping [50]. Continued innovation in phenotyping technologies and the exploitation of the wide genetic diversity found in local ecotypes and wild relatives, which serve as reservoirs of valuable traits, will be essential to overcome current limitations and support the growing economic and nutritional relevance of berry crops. Robust improvement of antioxidant traits will therefore depend on standardized phenotyping, multi-omics integration, and advanced modeling to disentangle genetic effects from environmental noise [209].

## 5. Conclusions

Berry crops are highly valued for their rich antioxidant capacity, which are vital for human health. Historically, breeding for antioxidants was indirect, often occurring as a byproduct of selection for environmental stress tolerance. However, driven by the increasing demand for functional foods, targeted breeding for enhanced antioxidant potential has become a priority.

The future of improving berry antioxidant capacity and climate resilience depends on a strategic shift toward targeted breeding, leveraging the genetic diversity of wild relatives and integrating advanced molecular tools such as QTL mapping, marker-assisted selection, genomic selection, and CRISPR/Cas9-based editing. These technologies enable the rapid introgression or modification of favorable alleles. When combined with multi-omics data (genomics, epigenomics, transcriptomics, proteomics, and metabolomics), they provide a comprehensive understanding of the gene regulatory networks and metabolic pathways that govern antioxidant biosynthesis and accumulation—both under optimal and stress-induced environmental conditions associated with climate change.

This holistic insight facilitates the development of biomarkers for selecting high-antioxidant, stress-tolerant genotypes and allows precise editing of key genomic regions to modify antioxidant metabolism, thereby accelerating the creation of cultivars with superior nutritional quality, resilience, and productivity. While targeted editing offers rapid genetic improvement, especially for species with long breeding cycles, several challenges persist, including low regeneration capacities, complex regulatory frameworks for gene editing across countries [210,211], the difficulty of translating complex omics data into practical breeding decisions, managing polygenic traits affected by fluctuating climates, and ensuring sufficient phenotyping capacity.

In addition to molecular techniques, agronomic practices, particularly seed priming and grafting, are also valuable for increasing the antioxidant content of berry crops. Despite these challenges, integrated molecular and agronomic strategies present significant opportunities to develop highly nutritious, climate-resilient berry cultivars that meet evolving consumer and sustainability demands.

## Figures and Tables

**Table 1:** Origins, major producer countries, and growing requirements of key berry crops and grapes according to FAOSTAT [5].

Crop	Scientific Name	Native Origin	Global Production	Main Producers	Soil & Climate Requirements
Raspberry	*Rubus idaeus*	Europe, Northern Asia	~940 kt	Russia (219 kt), Mexico (165 kt), Serbia (122 kt), Poland (118 kt), USA (100 kt)	Fertile, well-drained soils; cool winters for dormancy and flowering
Blueberry	*Vaccinium* spp.	North America	~1.1 million t (Mt)	USA (300 kt), Peru (230 kt), Canada (165 kt), Chile (125 kt), Spain (70 kt)	Acidic soils; consistent moisture; frost protection
Cranberry	*Vaccinium macrocarpon*	Northeastern North America	~470 kt	USA (300 kt), Canada (151 kt), Turkey (12 kt)	Acidic, water-saturated soils; benefit from regular field renewal
Grape	*Vitis vinifera*	Near East	>72.5 million t (Mt)	China (12.5 Mt), Italy (8.1 Mt), Spain 5.9 Mt), USA (5.4 Mt), France (6.2 Mt)	Wide soil tolerance; good drainage; warm, dry summers

**Table 2:** Genes analyzed for their functional involvement in the antioxidant capacity of blueberries.

Enzyme Class	Gene	Functional Role	Reference
Multidrug and Toxic Compound Extrusion Transporters (MATE)	*VcMATE2*	Facilitate anthocyanin movement across cellular membranes during ripening	Chen et al., 2015 [83]
	*VcMATE3*		
	*VcMATE5*		
	*VcMATE7*		
	*VcMATE8*		
	*VcMATE9*		
Glutathione S-transferases (GSTs)	*VcGSTF8*	Highly expressed during fruit ripening; strong correlation with anthocyanin accumulation	Ζhang et al., 2024 [84]
	*VcGSTF20*		
	*VcGSTF22*		
O-methyltransferases (COMTs)	*VcCOMT40*	Highly expressed during fruit development; involved in lignin biosynthesis and anthocyanin modification	Liu et al., 2021 [89]
	*VcCOMT92*		
Flavonoid Biosynthesis Enzymes	*VcCHS*	Initiates flavonoid biosynthesis; upregulated during ripening	Chu et al., 2024; Zhang et al., 2025 [90,91]
	*VcCHI*	Converts chalcone to naringenin; active in ripening fruit	
	*VcF3H*	Hydroxylates flavonoids contribute to anthocyanin diversity	
	*VcF3′H*	Adds hydroxyl group; modifies anthocyanin structure	
	*VcF3′5′H*	Adds hydroxyl groups for delphinidin-type anthocyanins	
	*VcDFR*	Converts dihydroflavonols to leucoanthocyanidins	
	*VcANS*	Synthesizes anthocyanidins; active during ripening	
	*VcUFGT*	Glycosylates anthocyanins for stability and vacuolar transport	
Flavonol Synthase (FLS)	*VcFLS* homologs	Produces flavonols like quercetin and kaempferol in fruit skin	Günther et al., 2020 [86]
Leucoanthocyanidin Reductase (LAR)	*VcLAR*	Synthesizes catechin; contributes to proanthocyanidin biosynthesis	
Anthocyanidin Reductase (ANR)	*VcANR1*	Produces epicatechin; active in fruit tissues	

**Table 3:** Genes analyzed for their functional involvement in the antioxidant capacity of raspberries.

Enzyme Class	Gene	Functional Role	Reference
Polyketide synthase (PKS)	*RiPKS1*	Encodes a typical chalcone synthase (CHS) active in naringenin production	Zheng et al., 2001 & Kassim et al., 2009 [109,110]
	*RiPKS2*	Characterized from raspberry cell cultures but found inactive due to specific amino acid exchanges	
	*RiPKS3*	Produced mainly p-coumaroyltriacetic acid lactone (CTAL)	
	*RiPKS5*	Catalyzes first step in flavonoid biosynthesis	Woodhead et al. (2010) [112]
4-coumarate:CoA ligase (4CL)	*Ri4CL1*	Catalyzes activation of 4-coumarate to CoA esters used in phenylpropanoid pathway	
	*Ri4CL2*		
	*Ri4CL3*		
Anthocyanidin synthase (ANS)	*RiANS*	Conversion of leucocyanidins to anthocyanidins	
Lipoxygenase (LOX)	*RiLOX*	Involved in lipoxygenase pathway	
Terpene synthase (TPS)	*RiTerpSynth*	Involved in monoterpene biosynthesis	
3-hydroxy-3-methylglutaryl-coenzyme A (HMG CoA) reductase	*ERubLR_SQ8.1_H09*	Involved in mevalonic acid pathway	
Isopentenyl-diphosphate delta-isomerase (IDI/IPI)	*ERubLR_SQ13.1_F09*		
Aconitase (ACO)	*ERubLR_SQ13.2_C12*		

**Table 4:** Genes analyzed for their functional involvement in the antioxidant capacity of blackberries.

Enzyme Class	Gene	Functional Role	References
Phenylalanine ammonia-lyase (PAL)	*RuPAL*	Converting L-phenylalanine to trans-cinnamic acid, providing precursors for flavonoids, lignin, and phenolic acids	Vogt et al., 2010 [126]
Chalcone synthase (CHS)	*RuCHS*	Catalyzes the formation of naringenin chalcone	Dao et al., 2011 [127]
Chalcone isomerase (CHI)	*RuCHI*	Catalyzes the stereospecific isomerization of chalcones into flavanones	Jez et al., 2000 [128]
Dihydroflavonol 4-reductase (DFR)	*RuDFR*	Reduces dihydroflavonols to leucoanthocyanidins	Petit et al., 2007 [129]
Anthocyanidin synthase (ANS)	*RuANS*	Oxidizes leucoanthocyanidins to anthocyanidins	Saito et al., 2013 [130]
UDP-glucose:flavonoid 3-O-glucosyltransferase (UFGT)	*RuUFGT*	Glycosylates unstable anthocyanidins at the 3-hydroxyl position	Kobayashi et al., 2001 [131]

## Data Availability

No new data were created or analyzed in this study.
